# Sextech Use as a Potential Mental Health Reprieve: The Role of Anxiety, Depression, and Loneliness in Seeking Sex Online

**DOI:** 10.3390/ijerph18178924

**Published:** 2021-08-25

**Authors:** Alexandra S. Marcotte, Ellen M. Kaufman, Jessica T. Campbell, Tania A. Reynolds, Justin R. Garcia, Amanda N. Gesselman

**Affiliations:** 1 The Kinsey Institute, Indiana University, Bloomington, IN 47405, USA; asmarcot@indiana.edu (A.S.M.); elkauf@iu.edu (E.M.K.); jesscamp616@ufl.edu (J.T.C.); tareyn@unm.edu (T.A.R.); jusrgarc@iu.edu (J.R.G.); 2 Luddy School of Informatics, Computing, and Engineering, Indiana University, Bloomington, IN 47405, USA; 3 Department of Psychology, University of New Mexico, Albuquerque, NM 87131, USA; 4 Department of Gender Studies, Indiana University, Bloomington, IN 47405, USA

**Keywords:** mental health, sextech, sexual behavior, depression, anxiety, loneliness

## Abstract

Depression, anxiety, and loneliness have long been recognized as global mental health concerns. To temporarily relieve psychological distress, self-soothing behavior is common, including engagement in sexual behaviors that are linked to positive mental well-being. Considering the COVID-19 pandemic further exacerbated many mental health ailments alongside physical distancing regulations, we specifically examined online sexual behavior via the use of emergent digital sexual technologies, or sextech. In a 2019 study of 8004 American adults, we assessed whether people experiencing higher anxiety, depression, and/or loneliness were more likely to engage in sextech use. Furthermore, we examined whether anxiety or depression mediated the association between loneliness and sextech use, as loneliness is one contributor to anxiety and depression. People with higher anxiety and depression were more likely to engage in sextech. However, those who were more lonely were less likely to engage with sextech, suggesting the aforementioned patterns were not due to lack of social connection. Our findings suggest people with mental health struggles may be drawn to interactive, digital forms of sexual behavior as a means of alleviating symptoms through distraction or self-soothing. This insight offers an important pathway for expanding the scope of mental health interventions, particularly as technology becomes increasingly prevalent and accessible in everyday life.

## 1. Introduction

In 2001, the World Health Organization (WHO) (Geneva, Switzerland) reported that one in four people around the world would be affected by a mental health disorder during their lifetimes [1]. At the time, depression was already the “fourth leading cause of the global disease burden” [2]. By 2010, a further rise in pervasiveness led WHO to deem depression a global crisis. Depression—along with other markers of mental health, including anxiety and psychological loneliness—has continued to increase in prevalence on an international scale, resulting in substantial concern and action from public health monitoring organizations [3]. Depression and anxiety affect more than 264 and 284 million people worldwide, respectively [4]. Furthermore, rates of comorbidity between anxiety and depression are high, ranging from 50% to 75% [5,6]. These high rates and degree of overlap has led to depression and anxiety serving as some of the most predominant mental health concerns investigated by researchers.

Depression and anxiety are also strongly linked with the experience of loneliness [7]. Psychological loneliness is conceptualized as a feeling of isolation and disconnection from others, and is robustly associated with an increased risk of negative affective conditions [8]. In a recent meta-analysis on depression, loneliness had a moderately significant effect, suggesting that loneliness may serve as a primary risk factor for depression [9]. Relatedly, in a large sample of over 15,000 individuals, more than half of the loneliest participants and nearly one-third of moderately lonely people were affected by depression [10]. Lonely individuals are also at increased risk for experiencing anxiety and suicidality. For example, longitudinal studies of undergraduate college students have demonstrated that loneliness predicts increased depression and anxiety over time [11].

Although these three forms of psychological distress—loneliness, anxiety, and depression—are robustly linked in the existing literature, sociodemographic variables also influence their prevalence and manifestations. Gender and sexual orientation appear to be especially influential. In terms of gender, studies have found that women, compared to men, are more sensitive to social contexts and desire more interpersonal connection [12]. As such, women are at greater risk of experiencing negative affective disorders such as depression and loneliness than are men and report more loneliness and depressive symptoms [13,14,15,16,17]. Sexual orientation similarly influences vulnerability to these affective disorders. Lesbian, gay, and bisexual (LGB) individuals report higher rates of depression and anxiety than their heterosexual peers [18,19]. Lack of social and political acceptance, as well as internalized homophobia are commonly cited as leading factors contributing to the high rates of depression and anxiety among LGB people [20,21]. Furthermore, bisexual individuals present with higher rates of depression and anxiety compared to lesbian and gay individuals [22,23]. With respect to loneliness among LGB individuals, connection to the LGB community is negatively associated with loneliness [24] and LGB individuals in rural areas and elderly LGB people are more likely to experience loneliness as they are less likely to have access to LGB communities [25,26]. These consistent patterns of results suggest that large-scale investigations of mental health should certainly target anxiety and depression but would be ineffective without also considering loneliness as an additional domain of mental health or as an exacerbating contributor to impaired mental health.

Absent treatment, individuals struggling with their own mental health may engage in a host of behavioral strategies to self-soothe or temporarily relieve psychological distress [27]. These relief strategies may include self-medication with drugs and alcohol, physical exercise, mindfulness exercises, seeking out social support from close others, or a host of other behaviors and activities that promote distraction, tranquility, or mood improvement [28,29,30,31,32,33]. In the context of loneliness, anxiety, and/or depression, many people are motivated to self-soothe in ways that involve turning toward others and engaging in social connection [34]. People are driven to seek close social connection as part of their inherent and fundamental need to belong [35], and feelings of distress ‘activate’ the attachment behavioral system [36]—an evolved psychological mechanism that serves to promote physical and psychological safety by motivating the ‘feeler’ to maintain close proximity with their ‘secure base’ (i.e., their close relationship partner). Thus, in times of mental duress like when experiencing depression or anxiety, people are likely to actively seek out support from other people in their close relationships.

Research on attachment theory and the attachment behavioral system has created a complex and detailed literature on how both children and adults seek out their secure base or attachment figure (i.e., close relationship partner) in times of need. In the great majority of these studies, seeking ‘close proximity’ meant being in the same physical space as one’s secure base. However, research on digital (i.e., online) socialization highlights how technologically-facilitated interactions can improve mental well-being [37]. For instance, in one study, emotionally distressed adolescents experienced a positive increase in mood after instant messaging with peers [38]. Technology-facilitated interactions provided a safe space in which adolescents could share their concerns with friends and ultimately served as a conduit for emotional relief.

The Internet provides a valuable avenue for researchers to reach those otherwise unreachable populations (e.g., people in rural areas, young adults, racially diverse samples), and is a seemingly useful tool for people to manage some aspects of their personal mental health. Although increased internet use (e.g., more hours spent on social media) is often associated with poorer mental well-being, particularly when utilized as a coping strategy [39], some aspects of online behavior may especially appeal to those struggling with mental health. In particular, online spaces provide a context in which people feel comfortable seeking content and connection due to relative anonymity, reduced stigmatization, affordability, and ease of access [40]. Additionally, according to the stimulation hypothesis, the Internet offers opportunities to stimulate—or enhance and grow—relationships [41,42]. Online environments can offer instantaneous social connection, which has been shown to temporarily relieve loneliness [43], offer a sense of connection [44], and provide stimulation that may relieve emotional and psychological discomfort on a short-term basis [45,46]. By examining potential outlets for psychological relief that may be accessed on a regular basis, we can better understand the diverse array of tactics people use to alleviate their mental health discomfort, even if only temporarily.

In the current paper, we examined the association between mental health concerns (i.e., depression, anxiety, loneliness) and online behavior. Specifically, we examined online sexual behavior. In particular, we focus on the use of emergent digital sexual technologies, or sextech, including sending sexually-explicit images or videos and visiting erotic webcam sites. Research has demonstrated that sexual satisfaction, both online and in-person, is strongly linked to mental well-being [47,48]. For example, in a daily diary survey of 349 both pre- and post-menopausal women, participants with higher (versus lower) sexual satisfaction were less likely to experience depression or anxiety over time [49]. Likewise, a longitudinal study of adolescent women showed that higher sexual well-being was significantly linked to lower self-reported depression and higher self-esteem over time [50]. Conversely, higher rates of depression and anxiety are linked to lower sexual satisfaction among men and women across the life course [51,52,53,54]. Furthermore, in a study of older women and men in 29 countries, researchers found a correlation between sexual well-being (defined as both physical and emotional sexual satisfaction as well as sexual health) and self-reported levels of happiness, suggesting that the connection between sexual satisfaction and happiness is largely universal [55]. Albeit a brief review, the literature amassed pinpoints sexuality and sexual satisfaction as key contributors to positive well-being.

Considering these physical and psychological health benefits, it follows that some people may wish to engage in sexual behavior as a method to promote positive feelings and to soothe negative affective symptoms. Researchers have identified hundreds of motivations for having sex [56]. The most common of these underlying motivations include a desire for social connection and mood improvement: seeking emotional intimacy, wanting to feel a connection, wanting to feel loved, wanting reassurance or approval, wanting to improve feelings about oneself or one’s self-esteem, along with simple desire for sexual gratification [57,58,59,60]. Further, some participants specifically report engaging in sex when they need relief from their feelings of stress [59,61,62]. People in romantic relationships also report engaging in sex as a tool for reducing attachment uncertainty, or anxiety around whether one’s partner will leave the relationship [56,63,64]. Engaging in sex for symptom relief does appear to be a more likely motivation for men than for women; however, both genders do report engaging in sex for tension relief or as a way to cope with stressful life circumstances [57,59,65]. Thus, individuals experiencing higher anxiety, depression, and/or loneliness may wish to seek out sexual outlets as a temporary solution to psychological distress. It is important to note, however, that depression, anxiety, loneliness, and other mental health issues have been linked to diminished sexual desire for some, suggesting that those experiencing these mental health difficulties might be less likely, on average, to pursue sexual activity across contexts than would people with fewer or less severe mental health struggles [66,67,68]. Nonetheless, those with anxiety, depression, and/or loneliness do experience sexual desire and engage in sexual behaviors to varying degrees [69].

The democratization of technology—and in particular, increased internet access—offers new avenues for seeking sexual fulfillment. The prevalence of online pornography use is well-documented; according to one nationally representative study from the United States, 46% of men and 16% of women reported accessing internet pornography in any given week [70]. Cross-cultural investigations suggest that 77% of college students across Sweden, Canada, Germany, and the United States have viewed online pornography at least once in their lifetimes [71]. Although online pornography is providing many with the opportunity for sexual gratification, the increasing popularity of emergent forms of sextech highlights the varied potential for pleasurable online interactions. Erotic webcam modeling (“camming”), for example, departs from more static forms of explicit online media. Camming offers users the ability to engage directly with performers, both as audience members of public shows and through one-on-one private interactions or messaging [72]. Similarly, virtual reality (VR) pornography offers users a more immersive experience than its two-dimensional counterpart [73]. Against the backdrop of the COVID-19 pandemic, these novel pathways for online sexual interactions have dramatically increased in popularity, with subscription-based platforms like OnlyFans expanding the visibility and diversity of opportunities for meeting one’s sexual and intimate needs [74,75].

Previous research has uncovered positive associations between online pornography consumption, loneliness, and depression [76,77], although perhaps most well-established are links between individuals’ self-perceived problematic pornography consumption and impaired mental health [78,79]. Although mental health correlates of online pornography use have been thoroughly examined, the associations between psychological well-being and novel, more interactive sextech behaviors, such as visiting cam sites or chatting with chatbots (software that simulates text-based communication with another person), remain under-examined and poorly understood.

Sextech use may be especially appealing to those with impaired mental health because they offer opportunities to form interpersonal connections relatively anonymously and with reduced stigma. Additionally, online sexual and social interactions may lessen the negative emotional experiences associated with mental health issues by providing stimulation that distracts from daily emotional challenges [72,73]. More specifically, sextech use may stimulate the release of dopamine, temporarily relieving psychological or emotional discomfort, which could prove particularly appealing for those experiencing anxiety and depression [80]. Indeed, early research suggested that those prone to depression may be especially likely to rely on online sexual activity to mitigate psychological symptoms (e.g., tension or high stress) [81,82]. In the case of anxiety, people may transfer their excitability to sexual contexts (known as excitability transfer) [83], particularly amongst those with lower inhibition to sexual responding [84]. Such inclinations may be further incentivized by the release of ‘feel-good’ neurotransmitters (e.g., dopamine, oxytocin), which could assuage needs for interpersonal contact or validation [73,85].

In summary, mental health difficulties—and specifically experiences with anxiety, depression, and loneliness—are widespread and represent a global public health imperative. This is especially true following the COVID-19 pandemic, as this environment likely created stress and tension for many in the form of employment changes, financial hardships, threats to personal health, and disconnection from in-person social support, among others. Although empirically supported therapies should continue to be recommended to those struggling with mental health difficulties, self-soothing for temporary symptomatic relief is widespread and thus warrants better understanding to more effectively intervene at the societal level.

Online sexual behavior may represent one such temporary relief strategy. As people’s sexual lives and technology have become increasingly intertwined, perhaps especially throughout the COVID-19 pandemic, we sought to examine online sexual behavior in the current investigation. Using a national sample of 8004 American adults, we investigated whether loneliness, anxiety, and/or depression were associated with sextech use (engagement with technologies offering sexual interaction or sexual content) via a moderated mediation model. Given that patterns of mental health differ across grander and sexual orientation, we included gender and sexual orientation as moderating variables of the associations between sextech use and each measure of mental health. Specifically, we sought to address the following research questions:

RQ1. Are higher levels of loneliness, anxiety, and/or depression associated with sextech engagement?
RQ2. Do anxiety and depression mediate the association between loneliness and sextech engagement?
RQ3. How do gender and sexual orientation moderate each of the aforementioned associations in RQ1 and RQ2?

## 2. Materials and Methods

### 2.1. Procedure

Data were collected in 2019 as part of a larger study of digital intimate life. The survey was sponsored by Docler Holding, LLC (Luxembourg City, Luxembourg), which owns LiveJasmin.com (accessed on 1 May 2021), an adult, live, interactive camming website. However, participants were not recruited or in any way drawn from the LiveJasmin platform. No prior power analyses were conducted.

### 2.2. Ethical Considerations

Data access and analysis procedures were approved by Indiana University’s Institutional Review Board (#1908475114).

### 2.3. Participants

The participants were 8004 American adults. To be eligible for participation, individuals had to be at least 18 years old, fluent in English, United States residents, and possess home internet access. Participants were recruited by Prodege^®^, (Los Angeles, CA, USA) using independent opt-in internet research panels for a population-based cross-sectional survey. Panelists were drawn from a diverse pool of established participants who have been continuously recruited over several years from a variety of venues, including mailings, referrals, corporate partnerships, and internet recruitment. Recruitment targeted demographic distributions (i.e., age, gender, ethnicity, region, income) reflected in the most recent US Current Population Survey. All data were collected online.

### 2.4. Measures

#### 2.4.1. Sociodemographics

Participants self-reported their age, gender (i.e., man, woman, non-binary (e.g., genderqueer, genderfluid), agender, another identity not listed, and do not know), sexual orientation (i.e., straight/heterosexual, homosexual/gay/lesbian, bisexual, and other), race/ethnicity (i.e., White, Black/African-American, South Asian [Indian, Pakistani, etc.], East Asian [Chinese, Japanese, etc.], North American Indian or Alaskan Native or Pacific Islander, and other); and relationship status (i.e., single and not seeing anyone, casually dating one or more people, in a committed relationship, engaged, married). See Table 1 for complete participant demographics and Table 2 for binary gender (male, female) and sexual orientation (heterosexual, homosexual) for reported sextech usage relevant to the present analyses.

#### 2.4.2. Mental Health

**Anxiety.** Participants completed the PROMIS Anxiety Measure Short Form 4a, a 4-item scale assessing the degree to which they experienced anxiety symptoms in the previous week (α = 0.92) [86]. An example item reads: “my worries overwhelmed me”. Responses ranged from never (1) to always (4). Higher scores represent higher levels of reported anxiety (see Table 2 for descriptive statistics of included measures).

**Depression.** Participants completed the Patient Health Questionnaire-9 (PHQ-9; α = 0.91) [87]. This 9-item scale measures depressive symptoms based on DSM-IV criteria for depressive disorders. An example item is, “little interest or pleasure in doing things”. Responses ranged from not at all (0) to nearly every day (3). Higher scores represent higher levels of reported depression.

**Loneliness.** We assessed loneliness using the 8-item UCLA Loneliness Scale (α = 0.87) [88]. An example item is, “I lack companionship”. Responses were made on 5-point scales ranging from never (1) to always (5). Higher scores represent higher levels of reported loneliness.

#### 2.4.3. Sexual Desire

To control for levels of sexual desire, participants responded to the following face-valid single item measure “How often do you experience sexual desire?” using a 7-point scale (1 = never, 7 = several times a day). Higher scores represent higher levels of reported sexual desire.

#### 2.4.4. Types of Sextech

We assessed frequency of sextech engagement across seven domains of sexual technology. Sex tech domains were generated by the researchers to provide a diverse array of sexual activities, including those offered by novel technologies: (1) sending sexually explicit images or videos (i.e., nudes, pictures of genitals) via chat or text; (2) visiting a camming website (e.g., Chaturbate); (3) participating in a live camming stream (e.g., tipping, posting messages for the performer); (4) using a coordinated teledildonic accessory with a partner (i.e., digitally connected toys that allow for remote sexual interaction) [89] (5) accessing virtual reality (VR) pornography; (6) playing sexually-explicit role-playing games (RPGs) or other video games online; and (7) exchanging sexually-explicit messages with a chatbot or other artificially-intelligent (AI) entity. Responses were made on 5-point scales from never (1) to always (5). A single item sextech use variable was constructed by summing the number of sextech domains used by each participant (*M* = 1.03, *SD* = 1.82).

## 3. Data Analysis

**Figure 1:**
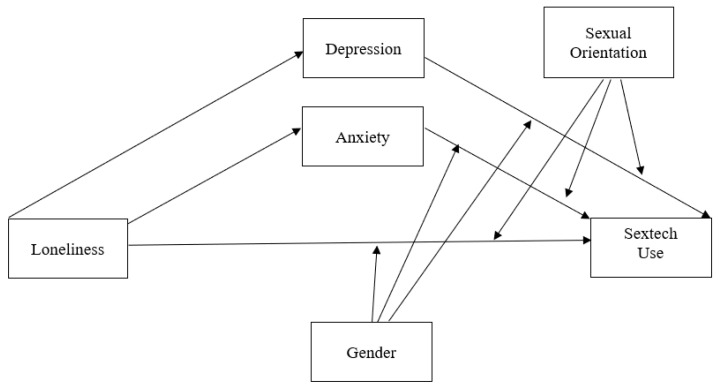
Proposed moderated mediation model. *Note.* Depression (M1) and anxiety (M2) will mediate the relationship between loneliness and sextech use. Additionally, gender (Q) and sexual orientation (V) will moderate the relationship between anxiety–sextech use and depression–sextech use.

We used Hayes’ [90] PROCESS version 3.5 macro for SPSS version 27 (IBM Corporation, Armonk, NY, USA) to test the moderated mediation model (see Figure 1). PROCESS investigates direct and indirect effects using a regression framework. We selected model 17, representing a mediation model that included moderators of the b and c paths. Our analysis used 5000 bias-corrected bootstrapped samples and 95% confidence intervals. Confidence intervals not including zero indicate significant indirect (i.e., mediated) effects. Further, the magnitude of the indirect effect is calculated by dividing the indirect coefficient by the direct coefficient to obtain a percentage of variance explained by the mediator.

Particularly helpful for tests of moderation, the PROCESS macro also probes interaction effects. These indicate whether the direct (i.e., non-mediated) or indirect (i.e., mediated) effects of X on Y are dependent on different levels of the moderator (e.g., whether loneliness is associated with sextech differently based on participants’ gender or sexual orientation). In our model, we simultaneously tested two mediators (i.e., depression, anxiety) and two moderators (i.e., gender sexual orientation) of the effect of loneliness on sextech. Three control variables were included in the model: sexual desire, relationship status, and age. Predictor variables and covariates were grand mean centered, following Cohen et al. [91].

## 4. Results

### 4.1. Descriptive Statistics: Frequency of Sextech Use

Descriptive statistics for sextech use and mental health variables are reported in Table 3. Sixty percent (60%) of the sample had never engaged in any of the seven assessed types of sextech. The most common form of sextech use was sending sexually explicit images or videos (i.e., “sexting”), with 30% of the sample reporting some level of prior engagement. Nearly one in five participants (18%) had visited a camming website, 14% had played a sexually-explicit RPG or video game online, 12% had participated in a camming stream, 11% had accessed VR pornography, 9% had used a coordinated teledildonic accessory, and 9% had exchanged sexually-explicit messages with a chatbot or AI entity. When assessing the summed variable of sextech use, participants had on average used at least one form in their personal lives (*M* = 1.03, *SD* = 1.82). Within the sample assessed, 79% of men and 51% of women reported using some form of sextech. Additionally, 61% of heterosexual and 83% of gay/bisexual participants reported using sextech.

### 4.2. Moderated Mediation

Our moderated mediation analysis revealed loneliness was significantly associated with sextech, dependent on gender and sexual orientation, but not in the expected direction. The direct effect was significant but negative, such that heterosexual men (*b* = −0.17, *t* = −4.16, *p* < 0.001, 95% CI = [−0.25, −0.09]), gay/bisexual men (*b* = −0.42, *t* = −5.19, *p* < 0.001, 95% CI = [−0.58, −0.26]), and lesbian/bisexual women (*b* = −0.28, *t* = −3.36, *p* < 0.001, 95% CI = [−0.44, −0.11]) who reported greater loneliness reported *less* engagement with the sextech domains. Among heterosexual women, however, loneliness was unrelated to their engagement with sextech (*b* = −0.02, *t* = −0.60, *p* = 0.55, 95% CI = [−0.10, −0.05]).

Loneliness was significantly and positively associated with both depression (*M*_1_; *b* = 0.50, *t* = 67.54, *p* < 0.001, 95% CI = [0.49, 0.52]) and anxiety (*M*_2_; *b* = 0.73, *t* = 64.54, *p* < 0.001, 95% CI = [0.70, 0.75]; paths *a*_1_ and *a*_2_). Participants who reported experiencing more loneliness also reported experiencing greater depression and anxiety.

The associations between depression (*M*_1_), anxiety (*M*_2_), and types of sextech used (Y) were moderated by gender and sexual orientation (paths *b*_1_ and *b*_2_). Reports of greater depression were associated with more sextech use for most participants. Both heterosexual men (*b* = 0.61, *t* = 9.98, *p* < 0.001, 95% CI = [0.49, 0.72]) and gay/bisexual men (*b* = 0.50, *t* = 4.44, *p* < 0.001, 95% CI = [0.28, 0.72]) who reported higher levels of depression also reported greater engagement with the various types of sextech. This pattern also emerged for heterosexual women (*b* = 0.27, *t* = 4.37, *p* < 0.001, 95% CI = [0.15, 0.39]) but to a lesser degree. Among lesbian/bisexual women however, depression was not significantly related to their sextech use (*b* = 0.16, *t* = 1.40, *p* = 0.16, 95% CI = [−0.06, 0.38]).

Anxiety (*M*_2_) followed a similar pattern. Increased anxiety was also associated with greater use of various types of sextech for male participants, including heterosexual men (*b* = 0.31, *t* = 7.50, *p* < 0.001, 95% CI = [0.23, 0.39]) and gay/bisexual men (*b* = 0.48, *t* = 6.25, *p* < 0.001, 95% CI = [0.33, 0.63]). However, the association among women differed depending on their sexual orientation. Lesbian/bisexual women who reported higher anxiety used more forms of sextech (*b* = 0.20, *t* = 2.65, *p* = 0.01, 95% CI = [0.05, 0.35]), but heterosexual women’s anxiety was unrelated to their sextech use (*b* = 0.03, *t* = 0.77, *p* = 0.44, 95% CI = [−0.05, 0.11]).

The indirect effect of loneliness on sextech use was not significant (*b* = −0.02, *t* = −0.60, *p* = 0.55, 95% CI = [−0.10, 0.05]). The effect of anxiety was non-significant in this model as well (*b* = 0.03, *t* = 0.77, *p* = 0.44, 95% CI = [−0.05, 0.11]). However, depression emerged as a significant mediating variable (*b* = 0.27, *t* = 4.37, *p* < 0.001, 95% CI = [0.15, 0.39]). Finally, Sobel tests revealed that depression fully mediated the association between loneliness and sextech use (*z* = 4.48, *p* < 0.001), but anxiety was not a significant mediator (*z* = 0.75, *p* = 0.48). See Table 4 for moderated mediation analysis results.

## 5. Discussion

In the current investigation, we leveraged a sample of over 8000 American adults to examine associations between facets of mental health and engagement with novel forms of sexual technology. Findings revealed that for most individuals, but not heterosexual women, greater loneliness with associated with reduced use of sextech. Put another way, those who were less lonely were more likely to use sexual technology. These patterns suggest that loneliness is not driving engagement with sexual technology, contradicting concerns that novel sexual technologies might be used to replace in person bonds [91]. Alternatively, these results may indicate that sextech is successfully offering an outlet for social connection, such that those who use these digital platforms experience reduced loneliness, consistent with the stimulation hypothesis [68,69].

Unlike loneliness, among most of our sample, increased anxiety (which was not significant among heterosexual women) and depression (which was not significant among non-heterosexual women) were each associated with heightened sextech use. These patterns may shed light onto the motivations behind pursuit of novel digital sexual domains. Our findings suggest that rather than replacing absent social connections, depressed and anxious individuals may turn to sexual technology for temporary symptom relief or distraction [72,73]. That is, use of these technologies may serve as a mitigation strategy for people struggling with psychological well-being, potentially offering temporary alleviation of symptoms via stimulation that distracts from daily emotional challenges, reduced stigma and/or concerns of discrimination inherent with in-person interactions, or the release of ‘feel good’ neurotransmitters. Indeed, recent research suggests novel forms of sexual technology (i.e., virtual reality pornography) effectively increase oxytocin levels in men [73]. Oxytocin is a neuropeptide that can promote wellbeing both through the stimulation of dopamine (which is implicated in reward processing) [92] as well as decrease stress [93]. Whether these novel forms of sexual technology effectively provide short or long-term reductions in psychological suffering is a promising avenue for future research.

The current findings suggest that individuals suffering from impaired mental health may use sextech as a tactic for self-soothing. However, results suggested that the associations between anxiety, depression, and engagement with sextech were not due to increased loneliness. These findings undermine the interpretation that depressed and anxious individuals seek digital sexual interactions because they are lonely in their offline lives. However, to the degree that loneliness leads to increased anxiety or depression (as was suggested by our data), lonely individuals may be more likely to engage with sexual technology when they experience these additional maladies. These patterns suggest future research would benefit from measuring anxiety and depression to discern whether any uncovered associations between loneliness and sextech use are not a result of other manifestations of mental distress. However, it may be the case that the distinctly remote nature of digital sexual behavior (i.e., the inherent lack of shared physical presence) residually exacerbates, rather than assuages, feelings of loneliness. Future researchers should consider this possibility when designing longitudinal investigations.

Despite its strengths, including a large demographically representative sample and emphasis on novel sexual technologies, the current study is not without limitations. First, data were correlational and collected at a single time point; thus, causal inferences cannot be drawn. Although findings are congruent with the interpretation that those who are depressed and anxious seek out digital sexual experiences, it is also plausible that seeking such experiences amplify mental distress. Indeed, prior work suggests online pornography consumption can contribute to depression at high frequencies or when the consumption is incongruent with one’s moral beliefs [94,95,96], which might extend to novel forms of sextech. Many of the types of sextech investigated here were more interactive than online pornography use, thus, it is possible they are more effective in alleviating distress because they more strongly simulate social interactions and social support. Indeed, one recent study found individuals watching virtual reality pornography felt more desired, more flirted with, and a stronger connection to the actors than those watching two-dimensional pornography [73]. To be sure, because our data were correlational, it cannot yet be ascertained whether sextech use is salubrious, and thus, implications for clinicians, educators, and marital therapists await experimental evidence.

Second, our data were self-reported. Given the sensitive nature of sextech use, participants may have under-reported engagement. Moreover, because the technological domains assessed here are relatively novel, prevalence may be less known, amplifying feelings of shame. However, our finding that sextech use was not linked to heightened loneliness may help reduce future stigma associated with these novel forms of sexual expression. Third, because engagement rates were relatively low, the seven technologies were combined into one composite measure. Future research might therefore examine each technology’s unique association with mental distress and efficacy for symptom alleviation. Last, our measure of sexual desire relied on a single item rather than an established scale to reduce participant burden. Although this item was face valid and only included as a covariate, whether the patterns uncovered here hold using established measures of sexual desire remains an open question for future work.

## 6. Conclusions

The advent of novel technologies, such as artificial intelligence and virtual reality, have broadened opportunities to pursue sexual fulfillment and social interaction. Academic research has struggled to keep pace with these burgeoning technological affordances. The current investigation sought to contribute to this lacuna by documenting how engagement with sextech corresponds to mental well-being. Our findings suggest those most strongly affected by anxiety and depression are turning to these novel venues, perhaps to distract from or self-soothe psychological distress. These patterns present a call for future work to examine the variegated consequences of these behaviors, from interpersonal to psychological health. Further, the recent COVID-19 pandemic has demonstrably heightened the demand for more focus on mental well-being for people of all demographics. In the United States, 40% of adults reported struggling with anxiety, depression, suicidal thoughts, and substance use as a consequence of the pandemic [97]. Worse still, increasing loneliness associated with COVID-19 lockdowns and social distancing requirements has been linked to intensified mental health symptomatology such as depression and anxiety [97,98,99]. Our findings suggest this mental anguish may amplify the appeal and prevalence of engagement with sexual technologies. As many countries around the world emerge after more than a year of sheltering due to the pandemic, a deeper understanding of mental health issues and potential mitigation strategies will likely prove critical for improving global public health. This knowledge may prove useful for designing effective interventions that can capitalize on the strengths of these novel technologies, such as their ease of access, enhanced anonymity, low STI risk, and widespread appeal.

## Figures and Tables

**Table 1:** Participant demographics.

	Have Used Sextech (*n* = 5192)	Have Not Used Sextech (*n* = 2812)	Total Sample (*n* = 8004)
**Age (Years)**			
Mean (*SD*)	41.05 (12.5)	49.68 (11.9)	44.08 (13.0)
**Gender identity *n* (%)**			
Male	3035 (58.5%)	792 (28.2%)	3827 (47.8%)
Female	2084 (40.1%)	2013 (71.6%)	4097 (51.2%)
Non-binary	60 (1.2%)	3 (0.1%)	63 (0.8%)
Agender	6 (0.1%)	3 (0.1%)	9 (0.1%)
Another Identity	6 (0.1%)	1 (0.0%)	7 (0.1%)
Don’t Know	1 (0.0%)	0 (0.0%)	1 (0.0%)
**Sexual orientation *n* (%)**			
Heterosexual	4180 (80.5%)	2675 (95.1%)	6855 (85.6%)
Homosexual/Gay/Lesbian	432 (8.3%)	90 (3.2%)	522 (6.5%)
Bisexual	506 (9.7%)	33 (1.2%)	539 (6.7%)
Other	74 (1.4%)	14 (0.5%)	88 (1.1%)
**Race/Ethnicity *n* (%)**			
White	3195 (61.5%)	1915 (68.1%)	5110 (63.8%)
Black/African American	748 (14.4%)	319 (11.3%)	1067 (13.3%)
South Asian	92 (1.8%)	26 (0.9%)	118 (1.5%)
East Asian	258 (5.0%)	139 (4.9%)	397 (5.0%)
North American Indian or Alaskan Native or Pacific Islander	63 (1.2%)	39 (1.4%)	102 (1.3%)
Hispanic or Latino	726 (14.0%)	296 (10.5%)	1022 (12.8%)
Other	110 (2.1%)	78 (2.8%)	188 (2.3%)
**Current relationship status *n* (%)**			
Single and not seeing anyone	1479 (28.5%)	814 (28.9%)	2293 (28.6%)
Casually dating one or more people	528 (10.2%)	66 (2.3%)	594 (7.4%)
In a committed relationship	796 (15.3%)	258 (9.2%)	1054 (13.2%)
Engaged	119 (2.3%)	32 (1.1%)	151 (1.9%)
Married	2270 (43.7%)	1642 (58.4%)	3912 (48.9%)

**Table 2:** Binary gender and sexual orientation descriptors of reported sextech usage.

	Have Used Sextech (*n* = 5192)	Have Not Used Sextech (*n* = 2812)	Total Sample (*n* = 8004)
**Gender identity and Sexual orientation *N* (%)**			
Heterosexual Male	2527 (48.7%)	763 (27.1%)	3290 (41.1%)
Heterosexual Female	1650 (31.8%)	1910 (67.9%)	3536 (44.5%)
Homosexual Male	239 (4.6%)	11 (0.4%)	250 (3.1%)
Homosexual Female	171 (3.3%)	79 (2.8%)	250 (3.1%)

**Table 3:** Descriptive statistics for study measures.

Variable	*M* ^1^	*SD* ^2^	Minimum	Maximum	α	Frequency (%)
Loneliness ^3^	2.50	0.86	1	5	0.87	
Depression ^4^	0.70	0.71	0	3	0.91	
Anxiety	2.10	1.05	1	5	0.92	
Sextech use ^5^	1.03	1.82	1	7	0.91	
Sexual desire ^6^	4.51	1.54	1	7		
Sending sexually explicit images or videos ^7^	1.52	0.94	1	5		2368 (29.6%) ^8^
Visiting a camming site	1.36	0.87	1	5		1481 (18.5%)
Participating in a live camming stream	1.23	0.71	1	5		930 (11.6%)
Coordinated teledildonic accessory usage	1.20	0.68	1	5		756 (9.4%)
Accessing virtual reality (VR) pornography	1.21	0.70	1	5		875 (10.9%)
Playing sexually-explicit role-playing games (RPG)	1.26	0.75	1	5		1108 (13.8%)
Exchanging sexually explicit messages with a chatbot/AI	1.18	0.64	1	5		704 (8.8%)

^1^*M* = mean; ^2^ *SD* = standard deviation. ^3^ Loneliness was measured on a 5-point scale, with higher scores representing greater loneliness. ^4^ Depression and anxiety were measured on a 4-point scale, with higher scores representing greater depression and anxiety. ^5^ Sextech Use is the sum of all sextech used by the individual participant. ^6^ Desire was measured on a 7-point scale, with higher scores representing greater desire. ^7^ All sexual behavior variables were measured on a 5-point scale, with higher scores representing more frequent engagement in that behavior. ^8^ Frequency of respondents who report using each form of sextech in any capacity.

**Table 4:** Moderated mediation analysis results.

	Coefficient	*SE*	*t*	*p*	95% CI
**IV to mediators (*a* paths):**					
Depression (*a*_1_)	0.50	0.01	67.54	<0.001	0.49, 0.52
Anxiety (*a*_2_)	0.73	0.01	64.54	<0.001	0.70, 0.75
**Mediators to DV (*b* paths):**					
Depression (*b*_1_)	0.27	0.06	4.37	<0.001	0.15, 0.39
Anxiety (*b*_2_)	0.03	0.04	0.77	0.44	−0.05, 0.11
**IV to DV (*c*′ path):**					
Loneliness (*c*_1_′)	−0.02	0.04	−0.60	0.55	−0.10, 0.05
**Moderators to DV (*c*′ paths):**					
Sexual Orientation (*c*_2_′)	0.47	0.06	8.09	<0.001	0.36, 0.58
Gender (*c*_3_′)	0.81	0.04	20.86	<0.001	0.73, 0.88
**Interactions:**					
Depression (*M*_1_) × Sexual orientation (*V*) (*b*_2i_)	−0.11	0.11	−0.95	0.34	−0.33, 0.12
Anxiety (*M*_2_) × Sexual orientation (*V*) (*b*_2i_)	0.17	0.08	2.22	0.03	0.02, 0.32
Depression (*M*_1_) × Gender (*Q*) (*b*_3i_)	0.34	0.08	4.09	<0.001	0.18, 0.50
Anxiety (*M*_2_) × Gender (*Q*) (*b*_3i_)	0.28	0.05	5.17	<0.001	0.17, 0.39
Loneliness (IV) × Sexual orientation (*V*) (*c*_4_′)	−0.25	0.08	−3.07	<0.001	−0.41, −0.09
Loneliness (IV) × Gender (*Q*) (*c*_5_′)	−0.15	0.06	−2.70	0.01	−0.26, −0.04
**Control Variables:**					
Sexual Desire	0.12	0.01	9.25	<0.001	0.09, 0.14
Relationship Status	0.00	0.04	0.01	0.99	−0.08, 0.08
Age	−0.03	0.00	−19.00	<0.001	−0.03, −0.02

## Data Availability

The data that support the findings of this study are available from the corresponding author upon reasonable request.
